# Strategies to identify medical patients suitable for management through same-day emergency care services: A systematic review

**DOI:** 10.1016/j.clinme.2024.100230

**Published:** 2024-07-19

**Authors:** Catherine Atkin, Rhea Khosla, John Belsham, Hannah Hegarty, Cait Hennessy, Elizabeth Sapey

**Affiliations:** a Birmingham Acute Care Research Group, Institute of Inflammation and Ageing, University of Birmingham, Birmingham B15 2GW, UK; b Department of Acute Medicine, University Hospitals Birmingham NHS Foundation Trust, Birmingham B15 2GW, UK; c The Medical School, University of Birmingham, Edgbaston, Birmingham, UK

**Keywords:** Acute medicine, Admission pathways, Same day emergency care, Scoring

## Abstract

Same-day emergency care (SDEC) in unplanned and emergency care is an NHS England (NHSE) priority. Optimal use of these services requires rapid identification of suitable patients. NHSE suggests the use of one tool for this purpose. This systematic review compares studies that evaluate the performance of selection tools for SDEC pathways.

Nine studies met the inclusion criteria. Three scores were evaluated: the Amb score (seven studies), Glasgow Admission Prediction Score (GAPS) (six studies) and Sydney Triage to Admission Risk Tool (START) (two studies). There was heterogeneity in the populations assessed, exclusion criteria used and definitions used for SDEC suitability, with proportions of patients deemed ‘suitable’ for SDEC ranging from 20 to 80%. Reported score sensitivity and specificity ranged between 18–99% and 10–89%. Score performance could not be compared due to heterogeneity between studies. No studies assessed clinical implementation.

The current evidence to support the use of a specific tool for SDEC is limited and requires further evaluation.

## Introduction

Effective delivery of acute care services requires rapid assessment and management of patients across emergency departments (ED) and acute medicine services, identifying those that can be safely discharged from hospital as well as those at risk of deterioration.

In the UK, same-day emergency care (SDEC), previously known as ambulatory emergency care (AEC), provides assessment and management of patients without overnight admission to an inpatient hospital bed.^1^ Consistent provision of SDEC has repeatedly been prioritised within NHS strategy,^2^^,^^3^ to reduce demand on inpatient services and avoid risks associated with inpatient admission.^4^

For unplanned medical attendances, SDEC is predominantly delivered within acute medicine services,^5^ accessed via referral from primary care and emergency medicine (EM) services.^1^ Acute medicine services vary considerably between hospitals nationally, including in the proportion of unplanned medical attendances assessed within SDEC, and the proportion discharged without overnight admission.^6^

Not all patients are suitable for SDEC, for example patients requiring urgent stabilisation or treatment only available as an inpatient; additionally, SDEC services often have limited capacity due to physical or workforce limitations. For efficient service delivery that maintains patient experience, only patients suitable for assessment within SDEC should be reviewed through this pathway. Using tools to help identify suitable patients, such as the Amb score,^7^ is recommended, but there is limited understanding of clinical performance of these scoring systems nationally.

This systematic review aimed to identify strategies (including scoring systems and selection criteria) currently used to identify medical admissions suitable for management within SDEC services, and to compare the populations in which they have been derived/validated and their ability to correctly identify suitable patients.

## Methods

The systematic review protocol was prepared using the Preferred Reporting Items for Systematic Reviews and Meta-Analyses (PRISMA) guideline^8^ and registered in the PROSPERO registry of systematic reviews (registration number CRD42022351082). Table 1 shows the inclusion and exclusion criteria. There were no language or publication date restrictions.

**Table 1:** Inclusion and exclusion criteria for the systematic review.

Inclusion criteria	Exclusion criteria
• All studies describing selection process for identification of patients presenting with acute medical problems suitable for review via ‘same day’ or ambulatory pathways. • Set within an acute hospital, with process or tool applied to unplanned medical admissions. • Observational and randomised studies; before and after implementation studies included. • Adults over the age of 16 years.	• No description of the selection process or tool applied to identify patients as suitable for assessment or treatment through ‘same day’/ambulatory pathways. • Not set in an acute hospital. • No assessment of medical admissions (eg studies assessing only surgical presentations or gynaecological presentations). • Studies of paediatric (<16 years) population only. • Systematic reviews.

Database searches were conducted in December 2023. The detailed search strategy is included in Supplementary Information. Search terms included both SDEC and AEC to reflect variation in naming conventions for SDEC-equivalent services. The search strategy was not designed to identify studies describing condition-specific ambulatory pathways, therefore these studies were excluded. Databases searched included the Cochrane Database of Systematic Reviews, MEDLINE (via Ovid), Embase (via Ovid), CINAHL Plus (via EBSCO), MEDLINE in Process (via Ovid), Cochrane Central Register of Controlled Trials (CENTRAL), PsycInfo (via Ovid), Healthcare Management Information Consortium database and Web of Science. Reference lists of included publications were also hand-searched.

In the UK, acute medical problems requiring secondary care assessment are managed through acute medicine services, with access via referral from other services. This is most commonly via referral from EM, following either initial triage or clinical assessment, but a proportion of patients are referred directly from primary care or community services, including general practitioners (GPs). Acute medicine services span multiple locations, with acute medical units (AMUs) providing care for patients requiring inpatient admission for at least one night, and SDEC delivering assessment, investigation and management of those requiring internal medicine services, but expected to be discharged without overnight admission.^6^ Studies that did not include acute presentations suitable for assessment by general or acute internal medicine clinicians were excluded. SDEC attendances are not classed as a hospital admission.^9^ Where studies described identification of patients for admission versus discharge in an acute medical setting, ‘discharge’ was assumed to include ‘same day’ management and these studies were included. Where studies described identification of patients for admission versus discharge in EDs, these were included if decision to discharge was equated with suitability for SDEC; studies were excluded if any patients with lengths of stay less than 12 h were classified as admitted.

### Study selection

Identified articles were exported into EndNote (Clarivate Analytics) for removal of duplicates. Abstracts were screened blindly and independently by two authors (CA and HH/CH/RK) within Rayyan (Qatar Foundation, Qatar) .^10^ Disagreements were resolved by full-text review with a third reviewer (JB).

After initial screening, two authors (CA and JB) reviewed full articles using predefined inclusion and exclusion criteria; disagreements were resolved by discussion with a third reviewer.

Data extraction using a standardised form (Supplementary Table 1) was performed by one reviewer (CA).

Risk of bias was assessed by one reviewer (CA), using the Prediction model Risk of Bias Assessment Tool (PROBAST).^11^

### Outcome measures

The primary outcome was selection tool performance in identifying patients suitable for management through SDEC (eg patients not requiring admission to an inpatient acute hospital bed). This included sensitivity, specificity, positive and negative predictive values of the tool in selecting suitable patients, and accuracy as assessed by the percentage of patients correctly identified, calculated as the proportion of true positives and true negatives from the sample population.

Secondary outcomes were readmission rates (unplanned reattendance within 7 or 30 days of discharge) and mortality (including 30-day mortality).

## Results

### Study selection

**Fig. 1:**
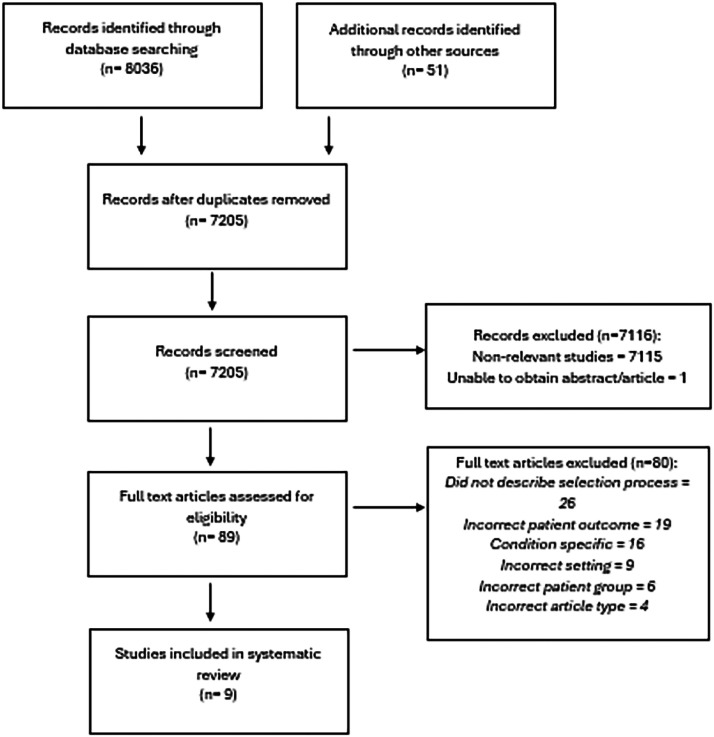
The article review process. PRISMA diagram for the systematic review.

Initial searches identified 8,036 abstracts. After removing duplicates, 7,205 abstracts were screened, with 89 selected for full-text screening. Nine met the inclusion criteria (Fig. 1).

Two studies described derivation and validation of a score, the Glasgow Admission Prediction Score (GAPS) ,^12^ and the Amb score (Ambs).^13^ Two studies assessed the Ambs alone.^14^^,^^15^ One study compared performance of the GAPS with triage nurse judgement.^16^ Two studies compared the Ambs and the GAPS,^17^^,^^18^ and two compared the Ambs, GAPS and the Sydney Triage to Admission Risk Tool (START).^19^^,^^20^ These scores as described in their original derivation studies are shown in Table 2.

**Table 2:** Scores evaluated in the included studies within this systematic review.

Amb score^13^		Glasgow Admission Prediction Score (GAPS)^12^		Sydney Triage to Admission Risk Tool (START) score^21^	
Age *<80* *≥80*	0 -0.5	Age	1 point per decade	Age (years) *16–19* *20–39* *40–59* *60–79* *≥80*	0 1 3 6 9
Not discharged from hospital within previous 30 days	1	Last admitted <1 year ago	5	Admission within previous 30 days	3
MEWS=0 *Yes* *No*	1 0	NEWS	1 point per point on NEWS score		
		Arrived in ambulance	5	Arrived in ambulance	4
		Triage category* 1 *2 (or 3+)* *3* >3	20 10 5 0	Triage category* 1 *2* *3* *4* *5*	24 16 11 5 0
Sex *Male*	-0.5				
Can take personal/public transport	2				
IV treatment not anticipated	2				
Not acutely confused	2				
		Referred by GP	5		
				ED arrival time *08:00–17:59* *18:00–07:59*	1 0
				Presenting problem *ENT/eye/head and neck* *Injury* *Cardiovascular* *Musculoskeletal* *Administrative* *Obstetrics, gynaecology* *Mental health* *Toxicological* *Skin, allergy* *Genitourinary* *Neurological* *Endocrine* *Respiratory* *General symptoms* *Social* *Abdominal/gastrointestinal* *Febrile illness* *Other medical*	-6 -4 -3 -3 -3 -3 -2 -2 -2 -1 -1 0 0 0 +1 +2 +3 +5

Score components as specified during original score derivation. MEWS: Modified Early Warning Score; NEWS: National Early Warning Score; IV: intravenous; GP: general practitioner; ED: Emergency Department; ENT: Ear, nose and throat. *GAPS uses Manchester triaging system, START uses Australasian Triage Scale

Table 3 outlines the characteristics of the included studies. Seven studies were conducted within the UK,^12^, ^13^, ^14^^,^^16^, ^17^, ^18^, ^19^ one in Italy^20^ and one in Malta.^15^ One study was available as conference abstract only.^19^ The number of patients included ranged from 54 to 322,846 (median 1,424 patients).

**Table 3:** Characteristics of studies included in the systematic review$.

Author	Study background	Design	Number enrolled	Patient selection	Inclusion	Exclusion	Age of participants	Gender (% male)	Frailty of participants	Comorbidities assessed
Ala 2012	Wales, single site(semi-rural, district general hospital), 4 weeks May–June 2010 (derivation); 3 weeks June–July 2011 (validation)	Observational; derivation and temporal validation, retrospective (derivation), prospective (validation)	625 (derivation: 282; validation: 343)	Random sample	Derivation: Medical emergencies referred by GP; Validation: Medical emergencies referred by GP or ED	Derivation: ED referrals, patients who died, self-discharges, admissions of 12–48 h; Validation: patients who died, self-discharges, admissions of 12–48 h	Derivation: Length of stay >48 h: mean 71.2 year (SD 16.2); length of stay <12 h: mean 57.2 (SD 17.5) Age ≥80 years: 23% Validation: Mean 66 years (SD 20) Age >80 years: 29%	Derivation: 36% Validation: 50%	Not reported	Coronary artery disease, heart failure, arrhythmia, diabetes, stroke/TIA, renal disease, chronic lung disease
Atkin 2022	Birmingham UK, single centre (urban, tertiary), 1 April 2019 – 9 March 2020	Retrospective, observational, external validation	7,365 episodes (6,848 patients) Ambs: 6,743; Ambs (NEWS2): 6,707; GAPS: 5,091; GAPS (NEWS2): 4,953	Consecutive	Patients seen by medical team, length of stay <48 h from hospital arrival; arrival to hospital between 08:00–16:59 Monday–Friday	Aged under 16, length of stay >48 h	Reported in 10-year age bands Age >80 years: 16.7%	42%	Not reported	Coronary artery disease, heart failure, arrhythmia, diabetes, stroke/TIA, renal disease, chronic lung disease
Burgess 2022$	Wales, single centre (urban, tertiary), Jan–December 2021	Retrospective, observational, external validation	350	NR	Patients seen by Older Persons Assessment Service (ED based service), aged >70 with frailty syndromes	None specified	Median 82.7 (IQR 77–90) All aged >70 years	39.7%	100% frailty service patients, mean CFS 5	Mean Charlson Comorbidity Index 6.5
Cameron 2015	Glasgow, UK, six units (three ED, two AMU, one minor injuries) in three hospitals (urban), 21 March 2010 – 20 March 2012	Retrospective observational, sample split for derivation and testing	322,846 (derivation: 215,231; testing: 107,615)	Consecutive	All unplanned attendances	Children, unknown age, unknown outcome, transfers, ‘irregular discharge’, cases with randomly missing data	Not reported	Not reported	Not reported	Not reported
Cameron 2017	Glasgow, UK, single site (urban, tertiary), 30 April 2014 – 16 May 2014	Prospective, observational	1,829	Consecutive	Emergency department attendances	<16 years, patients streamed to resus or minor injuries without formal nurse triage, left before treatment complete, incomplete data	Mean 47.3 (SD 21)	48.4%	Not reported	None
Cameron 2018	Glasgow/Sheffield, UK, two sites (urban, tertiary), 8–17 February 2016 (Sheffield) and 5–23 May 2016 (Glasgow)	Observational, external validation, prospective,	1,424 (787 Glasgow, 637 Sheffield)	Consecutive within time blocks	Emergency department attendances	<16 years, patients streamed to resus or minor injuries without formal nurse triage, left before treatment complete	Reported in 10 year age bands Age >80 years: 14%	49.2% (51.7% in Glasgow, 46.2% in Sheffield)	Not reported	Not reported
Dimech 2022	Malta, single centre (urban), Jan–March 2020	Observational, prospective	54	Random sample	Medical inpatient admission	Admitted to or reviewed by ITU, died during admission	Reported in 10 year age bands (under 30 s grouped) Age >80 years: 20%	53.7%	Not recorded (13% nursing home residents)	Not reported
Thompson 2015	Taunton, UK, single centre (tertiary)	Retrospective observational	200	Unclear	Medical patients referred to medical assessment unit	None specified	Not reported	Not reported	Not reported	Not reported
Salvato 2022	Italy, single site (urban), 30 September 2019 – 25 October 2019	Observational, external validation, prospective,	1,710	Consecutive when participating triage nurses available	Emergency department attendances	Paediatric patients; gynaecological, ‘fast track’ for specialist visit inc ENT, ophthalmology, dermatology, orthopaedics; left before disposition decision.	Median 54 years (IQR 34–74)	49%	Not reported	Not reported

$ Conference abstract only.

Five studies evaluated score performance in only medical patients,^13^, ^14^, ^15^^,^^17^ including one study that enrolled only a subset of medical patients within a specialist frailty service.^19^ One study assessed performance in a cohort combining patients presenting directly to acute medicine services, and patients presenting to the ED.^12^ Three studies evaluated patients within the ED,^16^^,^^18^^,^^20^ including but not restricted to medical patients.

No identified studies assessed implementation of a scoring system into clinical practice.

### Risk of bias and applicability

All included studies were assessed using the PROBAST tool (Table 4).^11^ Six studies were at risk of bias; however, this was limited to specific concerns within a maximum of two domains.^12^^,^^15^, ^16^, ^17^, ^18^^,^^20^

**Table 4:** Assessment of risk of bias and applicability of included studies.

	Risk of bias				Applicability			Overall	
	Participants	Predictors	Outcome	Analysis	Participants	Predictors	Outcome	Risk of bias	Applicability
Ala 2012^13^	**-**	**?**	**-**	**?**	**-**	**-**	**+**	**?**	**+**
Atkin 2022^17^	**-**	**-**	**-**	**+**	**-**	**-**	**+**	**+**	**+**
Burgess 2022^19^	**-**	**-**	**-**	**?**	**+**	**?**	**-**	**?**	**+**
Cameron 2015^12^	**-**	**?**	**+**	**-**	**+**	**-**	**+**	**+**	**+**
Cameron 2017^16^	**+**	**-**	**-**	**-**	**+**	**-**	**+**	**+**	**+**
Cameron 2018^18^	**+**	**+**	**-**	**-**	**+**	**-**	**+**	**+**	**+**
Dimech 2022^15^	**+**	**-**	**-**	**+**	**-**	**-**	**+**	**+**	**+**
Salvato 2022^20^	**+**	**-**	**-**	**-**	**+**	**-**	**+**	**+**	**+**
Thompson 2015^14^	**-**	**?**	**-**	**-**	**-**	**-**	**-**	**?**	**-**

Risk of bias and applicability assessed using the PROBAST tool.^11^ Classified in each domain as high risk (+), low risk (-) or unclear risk.

In four studies, the inclusion/exclusion criteria may have altered score performance compared to the intended clinical application, with three studies excluding patients requiring resuscitation or intensive care review,^15^^,^^16^^,^^18^ and one study not enrolling patients at times of increased service pressure.^20^ One study was at risk of bias in evaluation of predictors as the score component of ‘need for intravenous antibiotics’ (included in the Ambs) was assessed by a varied process that included decisions made later than where the score would be used in clinical practice.^18^ In three studies, it was unclear whether there was risk of bias in how predictors were assessed; in two studies, the method to determine ‘need for intravenous antibiotics’ was not described.^13^^,^^14^ Two studies had risk of bias in analysis,^15^ with differences in the proportion of missing data (and therefore participants included in analysis) when comparing scoring systems in one study.^17^

There were concerns regarding applicability in eight studies.^12^^,^^13^^,^^15^, ^16^, ^17^, ^18^, ^19^, ^20^ This related to the included participants in five studies; four studies included ED attendances not restricted to medical patients,^12^^,^^16^^,^^18^^,^^20^ and one study focused only on patients with frailty-related conditions.^19^ Six studies had applicability concerns regarding outcome; only two studies assessed the outcome of same-day discharge or discharge within 12 h compared to length of stay over 12 h,^14^^,^^19^ the remainder compared ‘admission’ without specified timeframe compared to discharge from hospital,^12^^,^^16^^,^^18^^,^^20^ length of stay <12 h compared to 12–48 h,^17^ <12 h compared to >48 h,^13^^,^^18^ or <24 h compared to >24 h.^15^

### Results of individual studies

Results of the individual studies included in the review are shown in Table 5.

**Table 5:** Results of the individual studies included in the systematic review.

Author	Outcome(s) assessed	Comparator	% with outcome	Tool(s) assessed (cutoff used)	Sensitivity (95% CI)	Specificity (95% CI)	PPV (95% CI)	NPV (95% CI)	AUROC (95% CI)	Other outcomes reported
Ala 2012	Length of stay <12 h	Length of stay >48 h	Derivation: 50.7% Validation: 33.5%	Validation: Ambs (≥5 = LoS <12 h)	96% (90–98%)	62% (55–68%)	*missing*	*Missing*	0.91 (0.88–0.94)	None
Atkin 2022	Length of stay <12 h	Length of stay 12–48 h	55%	Ambs ≥5	98.6% (98.1–98.9%)	11.4% (10.3–12.6%)	55% (53.8–56.2%)	87.8% (84.3–90.8%)	0.601 (0.588–0.614)	% correctly identified: 57%; readmission within 7 days 13.7% vs 5.8%, p 0.017; within 30 days 25.6% vs 13.6% p<0.001
				Ambs (with NEWS2) ≥5	98.8% (98.4–99.1%)	10% (9–11.1%)	54.5% (53.8–56.2%)	88.5% (84.7–91.6%)	0.612 (0.599–0.625)	% correctly identified: 56% readmission within 7 days 21.1% vs 6.4% p<0.001; readmission within 30 days 25.3% vs 13.8% p<0.001
				GAPS <16	50.4% (48.5–52.5%)	63.1% (61.3–64.9%)	51.4% (49.3–53.6%)	62.1% (60.3–63.9%)	0.608 (0.593–0.624)	% correctly identified: 57.5% readmission within 7 days 7.4% vs 5.1% p <0.005; readmission within 30 days 16.9% vs 10.7% p<0.005
				GAPS (with NEWS2) <16	50% (47.8–52.1%)	63.3% (61.5–65%)	50.5% (48.4–52.7%)	62.7% (60.9–64.5%)	0.606 (0.59–0.622)	% correctly identified: 57.5% readmission within 7 days 7.4% vs 5.2% p<0.005; readmission within 30 days 6.1% vs 4.5% P<0.001
Burgess	Same	Admission	77.7%	Ambs	18%	71%	30%	57%	0.67	None
2022^$^	day discharge			GAPS	25%	80%	85%	19%	0.61	
				START	26%	83%	86%	22%	0.61	
Cameron 2015	Discharge^	Admission^	NR	GAPS ≥16	81.7% (81.6–81.9%)^	78% (77.8–78.2%)^	85.7% (85.6–85.8%)^	72.5% (72.372.7%)^	Derivation: 0.8776 (0.8762–0.8791) Validation: 0.8774 (0.8752–0.8796)	% correctly identified: 80.3% (80.2–80.4%) 28 day readmission: 0.0933 increase in log odd readmission with per point
Cameron 2017	Discharge^	Admission^	40.7% admitted	GAPS (≤17=discharge)	86.6%^ (84.5–86.7%)	71.8%^ (68.4–75.0%)	81.7%*^	78.6%*^	0.876 (0.860 to 0.892)	Accuracy: 80.6% (78.7–82.4%) Triage nurse prediction: sensitivity 77.4%^ (74.8–79.9%), specificity 81.2%^ (78.2–84.0%), AUROC 0.875 (0.859 to 0.891), accuracy 79.0% (77.0–80.8%)
Cameron	Discharge^	Admission^	39.8% admitted,	GAPS (≤16 = discharge)	70.3%*	76%*	81.7%*	62.7%*	0.807 (0.785–0.83)	% correctly identified: 69.5%,
2018			60.2% discharged	Ambs	78.5%*	55.8%*	73%*	63.1%*	0.743 (0.717–0.769)	% correctly identified: 72.5%
				(pre-2012 version) (cut off ≥5= discharge)						Glasgow only: 42% admitted, GAPS AUROC 0.80; Ambs AUROC 0.724
										Sheffield only: 37% admitted, GAPS AUROC 0.817, Ambs AUROC 0.764
	Discharge within	Admission	Whole cohort: NR	GAPS	NR	NR	NR	NR	0.813 (0.789–0.837)	
	48hrs	>48hrs		Ambs					0.738 (0.709–0.767)	
	Discharge within	Admission	Whole cohort: NR	GAPS (<18)	NR	NR	NR	NR	0.841 (0.818–0.864)	
	12 h	>48 h		Ambs (>5)					0.769 (0.737–0.795)	
Dimech 2022	Discharge <24 h	Admission >24 h	20.4%	Ambs ≥5	90.9%*	67.4%*	41.7%*	96.7%*	NR	% correctly identified*: 72%
Thompson 2015	Discharge <12 h	Admission >12 h	21.6%	Ambs (including NEWS) ≥5	88%	69%	39%	95%	NR	% correctly identified*: 68%
	Discharge <12 h	Admission >12 h	NR	Ambs ≥5 (0900–1700 arrivals)	83%	56%	48%	NR	NR	
Salvato 2022	Discharge^	Admission^	18.1% admitted, 81.9% discharged	Ambs (≥5 = discharge)	71.5%^ (69.1–73.9%)	71.3%^ (65.9–76.3%)	91.8%^ (90.0–93.4%)	35.6%^ (31.9–39.6%)	0.77 (0.74–0.79)	Triage nurse predictive accuracy:
				GAPS (≤16= discharge)	51.1%^ (48.5–53.8%)	80%^ (75.1–84.3%)	92%^ (89.9–93.8%)	26.6%^ (23.8–29.6%)	0.72 (0.69–0.75)	Sensitivity 76.5% (71.3–81.1%), specificity 84.5% (82.5–86.4%), PPV 52.2% (47.5–56.9%), NPV 94.2% (92.7–95.4%),
				GAPS (cut off 17)	54.4%^ (51.8–57.1%)	78.4%^ (73.4–82.8%)	91.9%^ (89.8–93.7%)	27.6%^ (24.7–30.7%)	*Not reported*	AUROC 0.86 (0.84–0.89)
				START	23.6%^ (21.4–26.0%)	89.4%^ (85.4–92.6%)	52.2%^ (47.5–56.9%)	20.6%^ (18.4–22.8%)	0.61 (0.58–0.64)	
				START (cut off 20)	63.2%^ (60.6–65.7%)	53.2%^ (47.5–58.9%)	85.9%^(83.6–88.0%)	24.3%^(21.1–27.7%)	*Not reported*	

PPV: positive predictive value; NPV: negative predictive value; AUROC: area under receiver operating characteristic curve. *denotes measures calculated from data provided in study. ^to aid comparison, outcomes switched where reporting here to facilitate comparison of diagnostic accuracy measures.

### Score performance in medical patients alone Score derivation

Ala *et al* derived and internally validated the Ambs to predict likelihood of discharge within 12 h versus admission for over 48 h, at a single centre in Wales.^13^ Patients admitted for 12–48 h were excluded. Initial derivation included only patients referred directly from general practice; validation also included referrals from EM. Scores ≥5 identified patients likely to be discharged within 12 h, with sensitivity 96% and specificity 62%; PPV and NPV were not reported, and could not be calculated from the published data.

### Single score validation

Thompson calculated the Ambs retrospectively for 200 patients in a single acute medicine service.^14^ Limited information on patient demographics and selection process was reported; admissions via GP and ED were included. Diagnostic accuracy measures were reported for performance of the Ambs discriminating patients admitted for <12 h from those admitted for >12 h; 67.8% of patients were correctly identified. 39% with an Ambs suggesting suitability for SDEC were discharged in <12 h. Subgroup analysis restricted to those arriving between 09:00 and 17:00 demonstrated similar performance. Performance comparing ED and GP referrals was not reported.

Dimech *et al* calculated the Ambs for medical patients admitted to a single hospital in Malta, using information from inpatient records.^15^ Their primary aim was not to evaluate performance of the Ambs, but to identify the proportion of admissions potentially suitable for management through ambulatory pathways. Length of stay <24 h was used to indicate potential suitability for ambulatory management. Twenty percent of admissions were discharged within 24 h, of which 90% had an Ambs ≥5. An Ambs ≥5 was also found in 32.5% of those admitted for >24 h. Measures of diagnostic accuracy were not reported, but were calculated from the information provided.

### Comparison between scores

Atkin *et al* retrospectively evaluated performance of the Ambs and GAPS to identify patients suitable for SDEC, indicated by discharge within 12 h of hospital arrival.^17^ Analysis was restricted to patients arriving between 08:00 and 16:59 and discharged within 48 h. This single centre study evaluated the scores as originally described, and when substituting NEWS2 for MEWS/NEWS. The Ambs identified 57% of patients correctly; 55% with an Ambs suggesting SDEC suitability were discharged within 12 h. Ambs incorporating NEWS2 had similar performance (56% accuracy; 54.5% of ‘suitable’ patients admitted <12 h). Using a binary cut-off, the GAPS identified 57.5% of patients correctly; 51.4% with a GAPS suggesting SDEC suitability were discharged within 12 h. Incorporating NEWS2 had similar performance (accuracy 57.5%; 50.5% of ‘suitable’ patients admitted for <12 h). Although overall accuracy was similar for the Ambs and GAPS, the measures of diagnostic accuracy were significantly different (Table 5).

Burgess *et al* considered score performance in a single-centre retrospective analysis of patients seen through an ED-based SDEC-equivalent frailty service, restricted to adults aged >70 with ‘frailty syndromes’.^19^ Over 75% of included patients were discharged the same day. Comparison of the Ambs, GAPS and START in this cohort of selected frail patients demonstrated poor performance of all scores, with sensitivity of 18%, 25% and 26% respectively, suggesting that the scores did not correctly identify the majority of patients discharged without inpatient admission.

### Score performance in cohorts not restricted to medical patients

#### Score derivation

The GAPS was derived by Cameron *et al* in 2015.^12^ Retrospective data for all attendances to six units (three EDs, two medical assessment units and one minor injuries unit) were included, with a binary outcome of admission or discharge. Length of stay was not reported. A binary cut-off of more than 15 was used to indicate likely admission, predicting outcome with 80.3% accuracy overall, but the authors recommended adjusting cut-offs to local populations. Although the cohort included admissions directly to medical assessment units, score performance in this subgroup, or in medical patients specifically, was not reported.

#### Single score validation

Cameron *et al* compared performance of the GAPS with triage nurse prediction of admission versus discharge in patients presenting to ED triage.^16^ Score cut-off was adjusted to maximise sensitivity and specificity in the enrolled cohort. Triage nurse clinical judgement of likelihood of admission had similar accuracy to the GAPS.

#### Comparison between scores

Cameron *et al* (2018) compared performance of the GAPS and Ambs for patients triaged within the ED in a prospective study at two urban teaching hospitals.^18^ The Ambs as used in this study precedes the published validation (Supplementary Table 1). Score performance was assessed in predicting length of stay >48 h compared to discharge within 12 h, with optimum cut-offs to maximise sensitivity and specificity of GAPS<18 and Ambs >5. Cut-offs that maximised the proportion of correct predictions were GAPS <20 (74.2% correct, 95%CI 71.9–76.5%) and Ambs >5 (70.5% correct, 95%CI 68.1–72.9%). The AUROCs for score performance in predicting admission from the ED (vs discharge), and admission for >48 h (versus <48 h) were assessed (Table 5). Score performance in medical patients specifically was not reported.

Salvato *et al* compared performance of the Ambs, GAPS and START in identifying patients presenting to the ED that did not require admission, with score components recorded at ED triage and scores subsequently calculated by the research team.^20^ The triage scoring system used locally was mapped to that used in the GAPS and START. In their cohort, the Ambs performed better overall than the GAPS and START (assessed by AUROC) despite lower sensitivity. All three scores were outperformed by clinical judgement of trained triage nurses. Inclusion was not restricted to medical patients, and the proportion of patients presenting with medical problems or requiring admission to internal medicine, or score performance in these patient groups, was not provided.

#### Additional outcomes

Readmission rates were assessed in two studies (Table 5).^12^^,^^17^ The original GAPS study showed a positive relationship between GAPS and likelihood of subsequent hospital admission in those initially discharged from the ED.^12^ Atkin *et al* found an lower rate of readmission within 7 and 28 days in those with Ambs or GAPS suggesting suitability for SDEC.^17^

Mortality was not reported in any of the included studies.

#### Meta-analysis

Meta-analysis of the included studies could not be performed due to study heterogeneity.

## Discussion

This review describes the published evidence for criteria available to identify medical admissions suitable for SDEC. The included studies described and evaluated three scores: the Ambs,^13^ GAPS^12^ and START.^22^ These scoring systems vary in their components, but have some common factors, namely that increasing age and previous hospital admission reduce likelihood of same day discharge. Comparison of performance between scoring systems, or within different populations, was limited by variation in score definition, inclusion criteria, and outcome definitions.

The populations assessed within the studies varied considerably, with only one study including only medical admissions without exclusion criteria based on length of stay.^14^ The remainder enrolled medical patients within a larger cohort of ED admissions,^12^^,^^16^^,^^18^^,^^20^ medical admissions to a specialty SDEC service,^19^ or medical admissions meeting specified length of stay criteria.^13^^,^^15^^,^^17^ This will impact score performance, as the proportion of patients requiring hospital admission is different in diverse patient cohorts. Similarly, scores derivation and later assessment took place in different types of hospital, for example the Amb score was derived in a more rural setting, which likely has different logistical considerations when assessing suitability for SDEC care. The proportion of patients suitable for SDEC management varied considerably in the cohorts within the included studies, ranging from 20% to 80%.^14^^,^^20^

The outcome predicted by the scores in the included studies also varied. The GAPS and START aim to identify patients requiring admission compared to those that can be discharged from hospital,^12^^,^^22^ while the Ambs was intended to identify patients with length of stay less than 12 h, making them suitable for selection to an SDEC service.^13^ In the UK, SDEC attendances are not classified as an inpatient admission, and as such evaluations of scores that assessed a decision to admit versus discharge were included where the authors equated this to a decision that patients were suitable for management through SDEC.^9^ The START score was developed and validated in Australia and included patients admitted to observation wards with length of stay of 4–24 h classed as ‘admitted’.^21^^,^^22^ These studies therefore did not meet our inclusion criteria, as patients with lengths of stay suitable for SDEC were not differentiated from patients requiring admission. Similarly, Raita *et al* constructed machine learning models predicting the risk of hospitalisation at ED triage using retrospective data in the USA,^23^ but patients with medical presentations and a length of stay <24 h were included in the admitted patient cohort.

Comparing performance of the Ambs is also limited by changes in score definition used (Supplementary Table 2). Updated early warning scores have been substituted into the Ambs in some cases, accounting for changes in clinical practice.^24^ Substitution of score components may impact score performance, necessitating appropriate validation, particularly as NEWS2 incorporates acute confusion, which is itself a component of the Ambs. A preliminary version of the Ambs, used in one included study,^18^ was presented as a conference abstract prior to the Ambs derivation and validation discussed here.^13^ This variation suggests there may be variation in the ‘Ambs’ used in clinical practice, which may not have been appropriately validated.

Several studies restricted analysis to those patients where it was felt that scoring systems may be most useful as an adjunct to clinical judgement, where there is greatest uncertainty regarding need for hospital admission, by excluding patients within resuscitation or minor injuries areas.^16^^,^^18^ Score performance in these studies may underestimate performance if applied across the whole patient cohort.

Where score performance was compared to clinical judgement, clinician judgement was as or more accurate.^16^, ^20^ This suggests that the currently suggested scoring systems may not improve on judgement from appropriately experienced clinicians.

The search criteria for this review aimed to identify relevant studies describing a process used in the ED or acute medical setting to identify patients suitable for review within SDEC. It is possible that articles discussing ED disposition were not identified; however, no relevant articles were identified on hand-searching of included articles’ references.

Selection criteria for specific conditions were excluded from this review. Some conditions commonly managed through SDEC services, such as pulmonary embolism, have established criteria for ambulatory management.^25^ Studies assessing only patients with a diagnosis or suspected diagnosis of specific disease were excluded, as clinical judgement would be needed to determine the specific condition.

These results suggest that there has been no robust validation of performance of scoring systems to select medical patients suitable for assessment and management within SDEC. The variation in approach seen in the definitions of suitability for SDEC/ambulatory management suggests a lack of consensus regarding which patients are suitable for management within SDEC. Further evaluation is needed to understand the settings where scoring systems would be most beneficial, and could be practically incorporated, the cohort of patients these scores should aim to identify, and the acceptable accuracy of any scoring system. Current policy relies on a limited evidence-base; generating the in-depth understanding necessary to rapidly inform national policy will require a multi-centre study of factors assessing SDEC suitability, involving hospital sites across diverse settings. Components of the scoring systems previously evaluated should be assessed, including physiological observations and demographic factors, alongside other features potentially available at initial triage that could be incorporated into simple tools, such as mobility, functional measures, or symptom categories, with performance compared across settings. Assessment of performance should consider a wider range of clinically relevant outcomes, including unplanned readmission to hospital, alongside likely impact on staffing and infrastructure.

## Conclusion

Scoring systems (the Ambs, GAPS and START) have been suggested to identify patients suitable for management through SDEC services; however, evidence validating performance of these scores outside the setting in which they were derived is limited. There is considerable heterogeneity in how these scores have been evaluated, including in the definition of patients considered suitable for management through SDEC.

## Funding

CA has been funded by an NIHR Academic Clinical Lectureship. ES reports grant funding from the NIHR Midlands Patient Safety Research Collaboration, the NIHR Birmingham Biomedical Research Centre, NIHR Midlands Applied Research Centre, HDRUK Medicines Driver Programme and UKRI.

## CRediT authorship contribution statement

**Catherine Atkin:** Writing – review & editing, Writing – original draft, Project administration, Methodology, Investigation, Formal analysis, Data curation, Conceptualization. **Rhea Khosla:** Writing – review & editing, Formal analysis, Data curation. **John Belsham:** Writing – review & editing, Formal analysis, Data curation. **Hannah Hegarty:** Writing – review & editing, Formal analysis, Data curation. **Cait Hennessy:** Writing – review & editing, Formal analysis, Data curation. **Elizabeth Sapey:** Writing – review & editing, Formal analysis, Data curation, Conceptualization.

## Declaration of competing interest

The authors declare that they have no known competing financial interests or personal relationships that could have appeared to influence the work reported in this paper.

## Appendix Supplementary materials

Image, application 1
