# Supplementary material for: Strategies to identify medical patients suitable for management through same-day emergency care services: A systematic review
Source: Clin Med (Lond). 2024 Jul 19;24(4):100230. doi: 10.1016/j.clinme.2024.100230 (PMC11359741; doi:10.1016/j.clinme.2024.100230)
Supplement: Supplementary file 1 [file mmc1.docx]

**Supplementary information**

**Box 1:** Search strategy

| 1. (same day).ti,ab  2. (same-day).ti,ab  3. (SDEC).ti,ab  4. (1 OR 2 OR 3)  5. (ambulatory).ti,ab  6. (AEC). ti.ab  7. (5 OR 6)  8. (4 OR 7)  9. (score).ti,ab  10. (scoring).ti,ab  11. (select*).ti,ab  12. (criteria).ti,ab  13. (identif*).ti,ab  14. (9 OR 10 OR 11 OR 12 OR 13)  15. (acute medicine).ti.ab  16. (general medicine).ti.ab  17. (general internal). ti.ab  18. “EMERGENCY MEDICINE”/  19. (Emergency department).ti,ab  20.”EMERGENCY SERVICE, HOSPITAL”/  21. (Accident and emergency).ti,ab  22. (Acute medicine unit).ti,ab  23. (internal medicine).ti.ab  24. (acute care).ti.ab  23. (15 OR 16 OR 17 OR 18 OR 19 OR 20 OR 21 OR 22 OR 23 OR 24)  24. (8 AND 14 AND 23) |
| --- |

**Supplementary Table 1: Items included in data extraction form**

| **Study characteristics** | **Study results** |
| --- | --- |
| Title  Author  Publication year  Country  Study design  Prospective/retrospective  SIngle centre/multi-centre  Setting  Participant selection: consecutive or random  Participant selection: inclusion criteria  Paticipant selection: exclusion criteria  Recruitment dates  Tool(s) used  Cut-offs for tool(s) used  Outcome measures used  Definition of suitability for SDEC | Number of patients eligible  Number of patients included  Age of participants (median/mean, range)  Gender of participants  Frailty of participants  Comorbidity data collected?  Number of patients identified as suitable & unsuitable  Number of patients correctly identified as suitable & unsuitable  Sensitivity (95% CI)  Specificity (95% CI)  Positive predictive value (95% CI)  Negative predictive value (95% CI)  Percentage accuracy  AUROC (95% CI)  Reattendance  Mortality  Length of stay  Other outcome measures reported |

**Supplementary Table 2:** **Comparison of Amb score definitions used within included studies.**

| **Amb score (original derivation)**(13) | | **Amb score (2014)**(14,15) | | **Amb score (2022)** (17) | | **Preliminary Amb score**(18) | |
| --- | --- | --- | --- | --- | --- | --- | --- |
| Age  *<80*  *≥80* | 0  -0.5 | Age  *<80*  *≥80* | 0  -0.5 | Age  *<80*  *≥80* | 0  -0.5 | **Age**  ***<80*** | **1** |
| Not discharged from hospital within previous 30 days | 1 | Not discharged from hospital within previous 30 days | 1 | Not discharged from hospital within previous 30 days | 1 | Not discharged from hospital within previous 30 days | 1 |
| MEWS=0  *Yes*  *No* | 1  0 | **NEWS**  *0*  *≥1* | 1  0 | **NEWS2**  *0*  *≥1* | 1  0 | MEWS=0  *Yes*  *No* | 1  0 |
| Sex  *Male* | -0.5 | Sex  *Male* | -0.5 | Sex  *Male* | -0.5 | **Sex**  ***Female*** | **1** |
| Access to personal/public transport | 2 | Access to transport | 2 | Access to transport | 2 | **Access to personal/public transport** | **1** |
| IV treatment not anticipated | 2 | Will likely need IV treatment/access  Yes  No | 0  2 | IV treatment not anticipated | 2 | **IV treatment not anticipated by treating doctor** | **1** |
| Not acutely confused | 2 | Not acutely confused | 2 | Not acutely confused | 2 | **Not acutely confused** | **1** |

*Changes to definitions of score components or to point allocation highlighted in bold. MEWS: Modified Early Warning Score; NEWS: National Early Warning Score; IV: intravenous.*
